# Generation and Feasibility Assessment of a New Vehicle for Cell-Based Therapy for Treating Corneal Endothelial Dysfunction

**DOI:** 10.1371/journal.pone.0158427

**Published:** 2016-06-29

**Authors:** Naoki Okumura, Kazuya Kakutani, Ryota Inoue, Daiki Matsumoto, Tomoki Shimada, Makiko Nakahara, Yumiko Kiyanagi, Takehiro Itoh, Noriko Koizumi

**Affiliations:** 1 Department of Biomedical Engineering, Faculty of Life and Medical Sciences, Doshisha University, Kyotanabe, Japan; 2 Cell Science & Technology Institute, Inc., Sendai, Japan; Cedars-Sinai Medical Center; UCLA School of Medicine, UNITED STATES

## Abstract

The corneal endothelium maintains corneal transparency by its pump and barrier functions; consequently, its decompensation due to any pathological reason causes severe vision loss due to corneal haziness. Corneal transplantation is the only therapeutic choice for treating corneal endothelial dysfunction, but associated problems, such as a shortages of donor corneas, the difficulty of the surgical procedure, and graft failure, still need to be resolved. Regenerative medicine is attractive to researchers as a means of providing innovative therapies for corneal endothelial dysfunction, as it now does for other diseases. We previously demonstrated the successful regeneration of corneal endothelium in animal models by injecting cultured corneal endothelial cells (CECs) in combination with a Rho kinase (ROCK) inhibitor. The purpose of the present study was to optimize the vehicle for clinical use in cell-based therapy. Our screening of cell culture media revealed that RELAR medium promoted CEC adhesion. We then modified RELAR medium by removing hormones, growth factors, and potentially toxic materials to generate a cell therapy vehicle (CTV) composed of amino acid, salts, glucose, and vitamins. Injection of CECs in CTV enabled efficient engraftment and regeneration of the corneal endothelium in the rabbit corneal endothelial dysfunction model, with restoration of a transparent cornea. The CECs retained >85% viability after a 24 hour preservation as a cell suspension in CTV at 4°C and maintained their potency to regenerate the corneal endothelium in vivo. The vehicle developed here is clinically applicable for cell-based therapy aimed at treating the corneal endothelium. Our strategy involves the generation of vehicle from a culture medium appropriate for a given cell type by removing materials that are not favorable for clinical use.

## Introduction

The cornea serves as the window of the eye, and its transparency is critical for vision. One function of the corneal endothelium is the maintenance of corneal transparency, which is controlled by the regulation of aqueous humor flow to the corneal stroma by the pump and barrier functions of the corneal endothelium. Corneal endothelial cells (CECs) have very limited proliferative ability and rarely show mitosis in humans after birth [1–3]. The CECs continuously decrease in number at a rate of 0.6% per year throughout life [4], but this rate is sufficiently low to maintain the function of the corneal endothelium. However, severe damage to the corneal endothelium induces irreversible decompensation of endothelial function and leads to corneal haziness. Fuchs endothelial corneal dystrophies and decompensation following cataract surgery are the leading causes of corneal endothelial dysfunction [5].

Corneal transplantation is only therapeutic choice for treating corneal endothelial dysfunction [6]. Penetrating keratoplasty, which involves replacement of the full-thickness cornea, including the corneal endothelial layer, with a donor cornea has been performed since 1905 [6]. New surgical procedures have recently been introduced, including Descemet’s stripping endothelial keratoplasty (DSEK) and Descemet’s membrane endothelial keratoplasty (DMEK), where the diseased layer alone is selectively replaced instead of a full-thickness replacement. These procedures have undergone rapid development and have shown an explosive spread [7–11]. However, the problems associated with corneal transplantation, such as the shortage of donor corneas, the difficulty of the surgical procedure, and the incidence of graft failure in acute and chronic phases, have led researchers to devise new and less problematic strategies to provide less invasive and more effective therapy.

Regenerative medicine is now attracting researchers as a future innovative therapy for a number of diseases in many medical fields, including ophthalmology. For example, several groups have reported the successful transplantation of cultured corneal endothelial sheets in animal models [12–14]. However, the technical difficulty of transplanting a flexible sheet to the anterior chamber and the development of an artificial clinically applicable carrier are obstacles that limit corneal endothelial sheet transplantation. We have sought to overcome these obstacles through cell-based regenerative medicine. Our finding that a Rho kinase (ROCK) inhibitor enhanced the adhesion of cultured CECs to a substrate [15] suggested the possibility that ROCK inhibitors could be useful in cell-based therapy [16]. We used rabbit and monkey corneal endothelial dysfunction models to demonstrate the successful regeneration of corneal endothelium following the injection of cultured CECs in combination with a ROCK inhibitor [16].

In the current study, we conducted experiments to generate an optimized CEC vehicle for cell-based therapy aimed at treating corneal endothelial dysfunction. Our screening of various types of cell culture media revealed that RELAR medium promoted the cell adhesion property of CECs. We then used RELAR medium as a basis for generation of a cell therapy vehicle (CTV) by removing materials that are not favorable for clinical use. We then evaluated the feasibility of injecting CECs in CTV into a rabbit corneal endothelial dysfunction model, and we showed that the corneal endothelium was regenerated without any adverse effects.

## Materials and Methods

### Ethics statement

Human corneas were handled in accordance with the tenets set forth in the Declaration of Helsinki. Informed written consent in regard to eye donation for research was obtained from the next of kin of deceased donors. Donor corneas were obtained from SightLife^™^ (Seattle, WA). All tissue was recovered under the tenets of the Uniform Anatomical Gift Act (UAGA) of the particular state in which the donor consent was obtained and the tissue was recovered. The rabbit experiments were performed at Doshisha University (Kyoto, Japan) according to the protocol approved by Doshisha University Animal Experiment Committee (Approval No. A15011-2).

### Rabbit CECs Culture

Twenty rabbit eyes were purchased from the Funakoshi Co., Ltd. (Tokyo, Japan). The rabbit CECs (RCECs) were cultivated as described previously [16]. Briefly, Descemet’s membrane with RCECs was stripped and incubated in 0.6 U/mL of Dispase II (Roche Applied Science, Penzberg, Germany). The RCECs isolated from the Descemet’s membrane were seeded in 1 well of a 6-well plate coated with FNC Coating Mix^®^ (Athena Environmental Sciences, Inc., Baltimore, MD). The RCECs were cultured in a growth medium composed of Dulbecco’s modified Eagle’s medium (Life Technologies Corp., Carlsbad, CA) supplemented with 10% fetal bovine serum (FBS), 50 U/mL penicillin, 50 μg/mL streptomycin, and 2 ng/mL fibroblast growth factor 2 (Life Technologies Corp.). Cultivated RCECs were used at passages 1 through 3 for all experiments.

### Human CEC Cultures

Four human donor corneas (from persons >40 years of age) were used for human CEC (HCEC) cultivation, as described previously [17]. Briefly, Descemet’s membranes containing the HCECs were stripped from the corneas and the membranes were incubated with 1 mg/mL collagenase A (Roche Applied Science) at 37°C for 12 hours. The HCECs were then seeded in one well of a 48-well plate coated with laminin E8 fragments (iMatrix-511; Nippi, Incorporated, Tokyo, Japan) (2.0 μg/cm^2^) [17] [17]. The culture medium was prepared according to published protocols. First, basal medium was prepared, consisting of OptiMEM-I (Life Technologies Corp., Carlsbad, CA), 8% FBS, 5 ng/mL epidermal growth factor (Sigma-Aldrich Co., St. Louis, MO), 20 μg/mL ascorbic acid (Sigma-Aldrich Co.), 200 mg/L calcium chloride, 0.08% chondroitin sulfate (Wako Pure Chemical Industries, Ltd., Osaka, Japan), 50 μg/mL gentamicin, and 10μM SB431542 (Merck Millipore, Billerica, MA). This basal medium was then conditioned by culturing human bone marrow-derived mesenchymal stem cells (BM-MSCs) for 24 hours. Finally, the conditioned basal medium was collected for use as the culture medium for HCECs. Cultivated HCECs were used at passages 3 through 6 for the experiments.

### Evaluation of cell adhesion and survival

HCECs were seeded in the following media supplemented with 8% FBS at a density of 5.0×10^3^ cells/cm^2^ per well on a 96-well plate and cultured for 24 hours: OptiMEM-I, DMEM, RELAR medium (Cell Science & Technology Institute, Inc., Sendai, Japan), BME (Life Technologies Corp.), MEM (Life Technologies Corp.), MEMα (Life Technologies Corp.), M199 (Life Technologies Corp.), and F12/DMEM (Life Technologies Corp.). The effect of a ROCK inhibitor was evaluated by adding 100 μM Y-27632 (Wako Pure Chemical Industries, Ltd.) to the medium. RELAR medium is a modified type of RITC80-7 medium that contains supplemental serum substitutes, hormones and growth factors [18]. The CTV was prepared based on the composition of RITC80-7/RELAR medium by removing bioactive materials, such as hormones and growth factors and other possibly toxic materials. The CTV was generated and provided by Cell Science & Technology Institute, Inc. The composition of CTV is shown in S1 Table. Opeguard-MA Intraocular Irrigation Solution (Senju Pharmaceutical Co., Ltd., Osaka, Japan) and Ringer’s solution (Otsuka Pharmaceutical Co., Ltd., Tokyo, Japan) were used to provide a comparison with solutions that are approved for clinical use. The numbers of adhered HCECs on the culture plate were evaluated using the CellTiter-Glo^®^ Luminescent Cell Viability Assay and a Veritas^™^ Microplate Luminometer (Promega, Fitchburg, Wisconsin). Six samples were prepared for each group.

### Injection of CECs into a corneal endothelial dysfunction model

Twenty-one rabbits were used in this experiment. One eye of each rabbit was used and fellow eye was not used to avoid blindness. The rabbit corneal endothelial dysfunction model was created as described previously [16]. Briefly, the lens was removed to deepen the anterior chamber and the corneal endothelium was mechanically scraped from the Descemet’s membrane with a 20-gauge silicone needle (Soft Tapered Needle; Inami & Co., Ltd., Tokyo, Japan). A total of 5.0×10^5^ RCECs, suspended in 200 μl of Opeguard-MA Intraocular Irrigation Solution, DMEM, or CTV—all supplemented with 100 μM Y-27632 (Wako Pure Chemical Industries, Ltd.)—was injected into the anterior chamber of the corneal endothelial dysfunction model and the animals were kept in the face-down position for 3 hours under general anesthesia. As a control, cell-free CTV supplemented with 100 μM Y-27632 was injected (n = 3). The anterior segments were evaluated by slit-lamp microscopy with a Pentacam^®^ (OCULUS Optikgeräte GmbH, Wetzlar, Germany) instrument for 2 weeks. Corneal thickness was determined with an ultrasound pachymeter (SP-2000; Tomey, Nagoya, Japan), and the mean of 10 measured values was calculated (up to a maximum thickness of 1200 μm, the instrument’s maximum reading). Intraocular pressure was determined with a Tonovet^®^ (icare Finland, Vantaa, Finland) instrument. The corneal endothelium was evaluated by contact specular microscopy (Konan scanning slit specular microscope, Konan Medical, Nishinomiya, Japan).

Corneal endothelium regeneration by preserved RCECs was evaluated in the rabbit corneal endothelial dysfunction model (n = 3), and anterior segments were evaluated for 2 weeks. The preserved RCECs were prepared after harvesting from a culture plate by treatment with 0.05% Trypsin-EDTA (Life Technologies) for 5 minutes at 37°C. A total of 5.0×10^5^ RCECs were suspended in 100 μl of CTV and preserved for 24 hours at 4°C. A 100 μl volume of CTV, supplemented with 200 μM of Y-27632, was then added and the preserved RCECs were gently mixed by pipetting to obtain a preparation of 5.0×10^5^ RCECs suspended in 200 μl of CTV including Y-27632 (final concentration; 100 μM) for cell injection into the rabbit model. Cell-free CTV supplemented with 100 μM of Y-27632 was injected as control (n = 3). The ability of HCECs to regenerate corneal endothelium was evaluated by injecting 1.0×10^6^ HCECs (suspended in 200 μl CTV supplemented with 100 μM Y-27632) into the rabbit corneal endothelial dysfunction model (n = 3), and corneal specimens were evaluated after 48 hours.

### Staining

Rabbit corneal specimens were fixed in 4% formaldehyde and incubated for 30 minutes in 1% bovine serum albumin (BSA) to block nonspecific binding. Samples were incubated overnight at 4°C with antibodies against Na^+^/K^+^-ATPase (1:300, Upstate Biotechnology, Lake Placid, NY), ZO-1 (1:300, Life Technologies Corp., Carlsbad, CA), and N-cadherin (1:300, BD Biosciences, San Jose, CA). Alexa Fluor^®^ 488-conjugated goat anti-mouse (Life Technologies) was used as a secondary antibody at a 1:1000 dilution. Cell morphology was evaluated after actin staining with a 1:400 dilution of Alexa Fluor^®^ 594-conjugated phalloidin (Life Technologies). Nuclei were stained with DAPI (Dojindo Laboratories, Kumamoto, Japan). The samples were examined with a fluorescence microscope (TCS SP2 AOBS; Leica Microsystems, Wetzlar, Germany).

### Statistical analysis

The statistical significance (*P*-value) for mean values in two-sample comparisons was determined with the Student’s t-test. The statistical significance of comparisons of multiple sample sets was analyzed with Dunnett’s multiple-comparisons test. Results were expressed as mean ± SEM.

## Results

### Generation of the CEC injection vehicle

**Fig 1:**
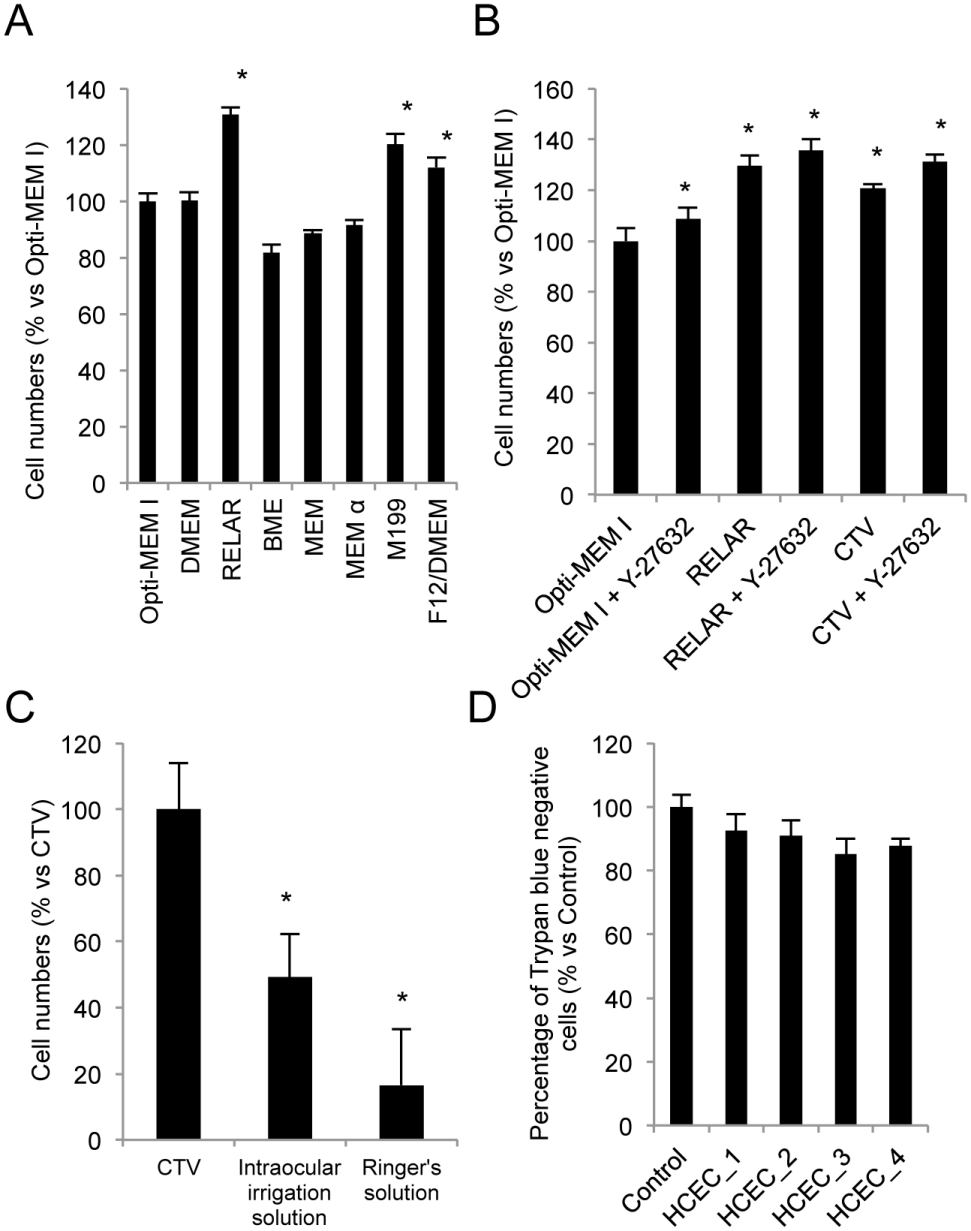
Generation of a human corneal endothelium cell (HCEC) vehicle for cell therapy. (A) Effect of various media types on HCEC adhesion to the substrate. HCECs were seeded with media without supplementation with FBS, and then the numbers of adhering cell were evaluated after 24 hours. RELAR, M199, and F12/DMEM media significantly enhanced cell adhesion when compared to OptiMEM-I. *p<0.01. (B) The cell therapy vehicle (CTV) was generated based on RELAR medium but with removal of materials such as hormones, growth factors, and other materials with possible toxicity. The numbers of adhered cells increased by 120.7% with CTV when compared to OptiMEM-I, although cell adhesion tended to be lower than with RELAR. However, HCECs seeded with the Y-27632 ROCK inhibitor showed a similar level of cell adhesion in RELAR and CTV (135.6% and 131.1% vs OptiMEM-I without Y-27632, respectively). *p<0.01. (C) The numbers of adhered HCECs were significantly lower in liquid Opeguard-MA Intraocular Irrigation Solution (used for intraocular surgery) and Ringer’s solution (used for intravenous drip infusion) than with CTV. *p<0.01. (D) Cell viability of HCECs was evaluated by Trypan blue staining after preservation in CTV for 24 hours at 4°C. HCECs derived from 4 independent donors and preserved in CTV showed showed a greater than 85% exclusion of Trypan blue. All experiments were performed in at least triplicate.

We screened the effect of various cell culture media on HCEC adhesion compared with adhesion in OptiMEM-I, a standard culture medium used for HCEC culture by several researchers, including us [17, 19]. HCEC adhesion was significantly enhanced in RELAR, M199, and F12/DMEM media (Fig 1A). RELAR medium was then further examined to test its applicability as a vehicle for cell injection therapy for corneal endothelial dysfunction.

RELAR medium was originally developed for serum-free culture of human renal proximal tubular epithelial cells. It is a modified RITC80-7 medium, additionally supplemented with some serum substitutes, hormones and growth factors. Hormones and growth factors, and especially mixtures of these, are not favored for clinical use if they are not necessary. Therefore, we removed those materials from the medium to generate the CTV. Cell adhesion was promoted by 120.7% by CTV, while it was promoted by 129.7% in comparison to OptiMEM-I. However, when HCECs were seeded with a ROCK inhibitor, the numbers of adhered cells were almost same in RELAR medium and in CTV (135.6% and 131.1%, respectively) (Fig 1B). The effects of representative liquids allowed for use in humans were also evaluated: Opeguard-MA Intraocular Irrigation Solution that is used for intraocular surgery and Ringer’s solution that is used for intravenous drip infusion. The numbers of adhered HCECs were significantly lower in these clinically used liquids than with CTV (Fig 1C).

The ability to preserve HCECs for a certain time is critical for transportation of HCECs from a cell culture facility to the operating room in a clinical setting. Therefore, we assessed cell viability of the HCECs after preservation in CTV for 24 hours at 4°C. All four lots of HCECs from independent donors exhibited viability higher than 85% (Fig 1D).

### RCEC injection using CTV as a vehicle in the rabbit corneal endothelial dysfunction model

**Fig 2:**
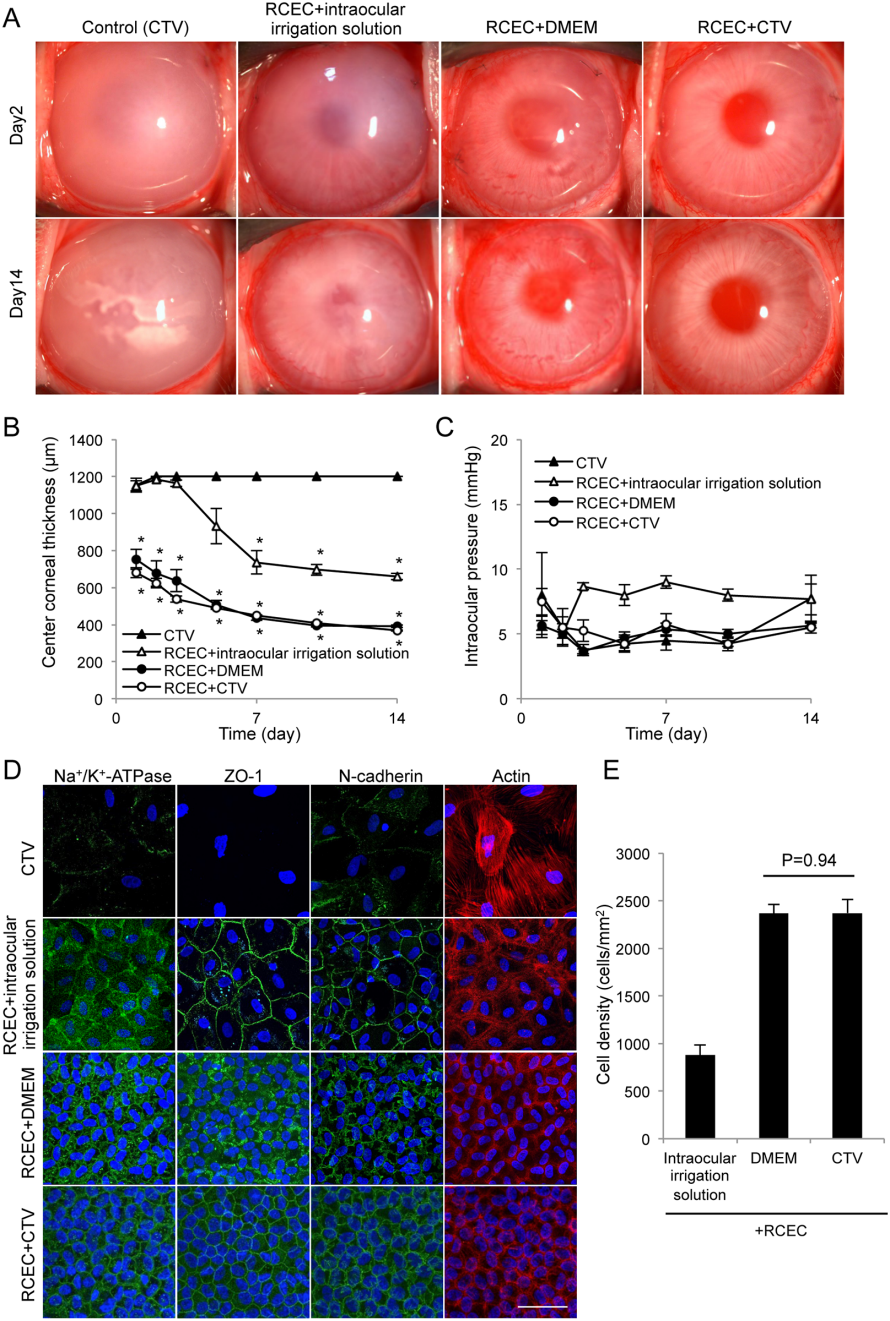
Rabbit corneal endothelium cell (RCEC) injection with cell therapy vehicle (CTV) enables regeneration of corneal endothelium. (A) A total of 5.0×10^5^ RCECs, suspended in 200 μl of intraocular irrigation solution, DMEM, or CTV supplemented with 100 μM Y-27632 was injected into the anterior chamber of the rabbit corneal endothelial dysfunction model (n = 3). CTV (200 μl) supplemented with 100 μM Y-27632 was injected into the anterior chamber of the rabbit corneal endothelial dysfunction model as a control (n = 3). Corneal transparency was restored by intracameral injection of RCECs suspended in either DMEM or CTV, while control eyes exhibited hazy corneas due to corneal endothelial dysfunction. (B) Central corneal thickness was evaluated with an ultrasound pachymeter and was restored to almost a normal value in the eyes transplanted with RCECs in DMEM or CTV. Eyes injected with RCECs in intraocular irrigation solution showed a thicker central corneal thickness when compared to eyes injected with RCECs in DMEM or CTV. (C) Intraocular pressure (IOP) elevation due to formation of cell aggregates in the eye is a possible complication and was evaluated with a Tonovet^®^. The IOP remained in the normal range throughout the 2 weeks of the study in all groups. (D) Regenerated corneal endothelium was evaluated by immunofluorescent staining 2 weeks after cell transplantation. The function-related markers Na^+^/K^+^-ATPase (pump function), ZO-1 (tight junction), and N-cadherin (adherent junction) were expressed in all regenerated CECs in eyes from both the DMEM and CTV groups. Actin staining showed hexagonal regenerated corneal endothelial cells. By contrast, control eyes had few fibroblastic transformed cells and lacked expression of the function-related markers. Scale bar: 50 μm. (E) Cell density of regenerated corneal endothelium formed by injecting RCECs was the same for cells suspended in DMEM and CTV, while cells suspended in intraocular irrigation solution showed lower cell density (Fig 2E).

We conducted experiments using the rabbit corneal endothelial dysfunction model to evaluate the feasibility of using CTV as a vehicle for CEC transplantation. Control eyes showed hazy corneas due to corneal endothelial dysfunction. Injection of RCECs in Opeguard-MA Intraocular Irrigation Solution as an injection vehicle resulted in a slightly less hazy cornea than the control but a transparent cornea was not regenerated. This suggested that an injection vehicle, rather than an intraocular irrigation solution used in eye surgery, would be required for cell-based therapy. We previously reported [16] that corneal transparency was restored in the endothelial dysfunction model by intracameral injection of RCECs suspended in DMEM supplemented with Y-27632. Similarly, in the present case, intracameral injection of RCECs suspended in CTV supplemented with Y-27632 restored corneal transparency (Fig 2A). The central corneal thickness, which is one of the critical clinical indicators of corneal endothelial health, was restored to an almost normal value in the eyes in which RCECs were transplanted using DMEM or CTV (Fig 2B).

The IOP was also examined to check for possible elevation due to formation of cell aggregates in the eye as a possible complication. The IOP remained in the normal range throughout the 2 weeks of the study in all groups (Fig 2C). Immunofluorescent staining demonstrated that the function-related markers Na^+^/K^+^-ATPase (pump function), ZO-1 (tight junction), and N-cadherin (adherent junction) were expressed along the cell cortex in all regenerated CECs in eyes in both the DMEM and CTV groups. Actin staining revealed that the regenerated corneal endothelium was a sheet-like structure of hexagonal cells. The control eyes had a few fibroblastic transformed cells with no expression of the function-related markers and the intraocular irrigation solution group had a lower density of cells with only partial expression of the function-related markers (Fig 2D). The cell density of the regenerated corneal endothelium was similar in the eyes injected with RCECs in DMEM or in CTV (Fig 2E).

### Feasibility of RCEC preservation using CTV

**Fig 3:**
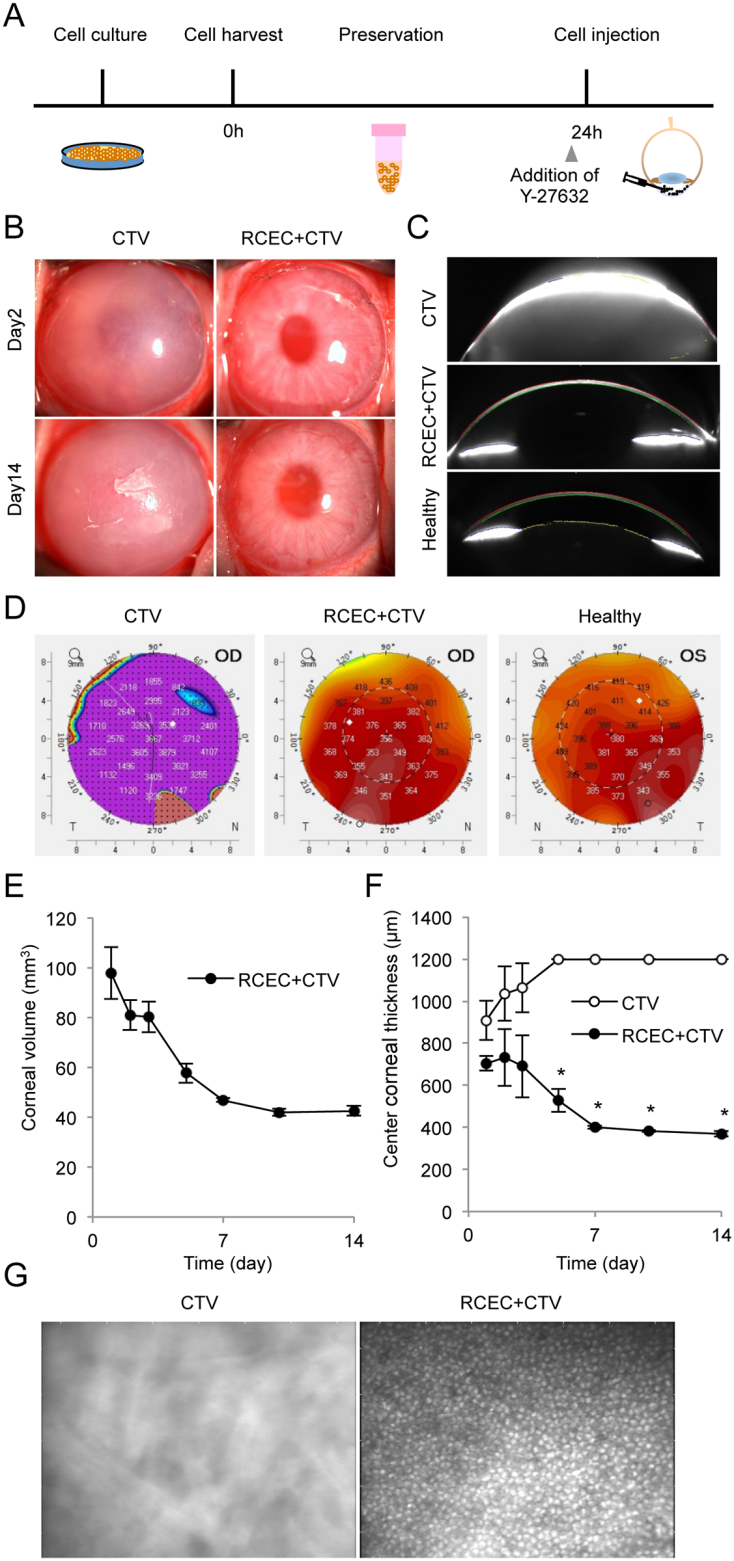
Rabbit corneal endothelium cells (RCECs) preserved in CTV regenerate corneal endothelium. (A) Schematic image showing the protocol for assessment of the feasibility of cell preservation in CTV. RCECs were harvested from the culture plate and suspended in CTV for 24 hours at 4°C, the ROCK inhibitor, Y-27632, was added to the RCECs before injection, and RCECs were injected into the anterior chamber of the rabbit corneal endothelial dysfunction model (n = 3). As a control, CTV supplemented with Y-27632 was injected into the rabbit corneal endothelial dysfunction model (n = 3). (B) Slitlamp microscopy showed that a transparent cornea was formed by the preserved RCECs, while the control eyes injected with vehicle alone exhibited hazy corneas. (C) Scheimpflug images showed the restoration of an anatomically normal cornea similar to a healthy cornea following injection of preserved RCECs. Control eyes injected with vehicle only exhibited corneal edema due to corneal endothelial dysfunction. (D) Corneal thickness was evaluated with a Pentacam^®^. The color map showing corneal thickness demonstrated that eyes injected with preserved RCECs exhibited normal thicknesses throughout the center to the periphery, whereas control eyes exhibited thick corneas after 2 weeks. (E) Corneal volumes evaluated with a Pentacam^®^ showed normal levels after RCEC injection. (F) Central corneal thickness evaluated with an ultrasound pachymeter showed that eyes injected with preserved RCECs had corneas of normal thickness. (G) Regenerated corneal endothelium was examined in vivo by contact specular microscopy. A hexagonal monolayer sheet structure was observed in the eyes injected with preserved RCECs, whereas no similar structure was observed in the control eyes.

We evaluated whether RCECs preserved for 24 hours would still be useful for transplantation. The RCECs were harvested from the culture plate and suspended in CTV for 24 hours at 4°C. Y-27632 was then added to the RCECs and they were injected into the anterior chamber of the rabbit corneal endothelial dysfunction model (Fig 3A). Slitlamp microscopy showed that the eyes transplanted with preserved RCECs developed transparent corneas, while control eyes injected with vehicle exhibited hazy corneas (Fig 3B). Scheimpflug images obtained with a Pentacam^™^ instrument showed the successful regeneration of an anatomically normal cornea similar to a healthy cornea by RCEC injection, whereas the control eyes showed corneal edema due to corneal endothelial dysfunction (Fig 3C). A color map of corneal thickness demonstrated that preserved RCECs regenerated normal corneal thickness from the center to the periphery of the cornea, while the control eyes showed thick corneas (Fig 3D). The corneal volume was normal level after injection of preserved RCECs (Fig 3E). The central corneal thickness, evaluated with an ultrasound pachymeter, was normal after 1–2 weeks in the eyes injected with preserved RCECs (Fig 3F). A representative image of regenerated corneal endothelium examined by contact specular microscopy showed a hexagonal monolayer sheet structure, but no image was obtained in the control eyes (Fig 3G).

### Histological assessment of the corneal endothelium regenerated by RCECs and HCECs after preservation

**Fig 4:**
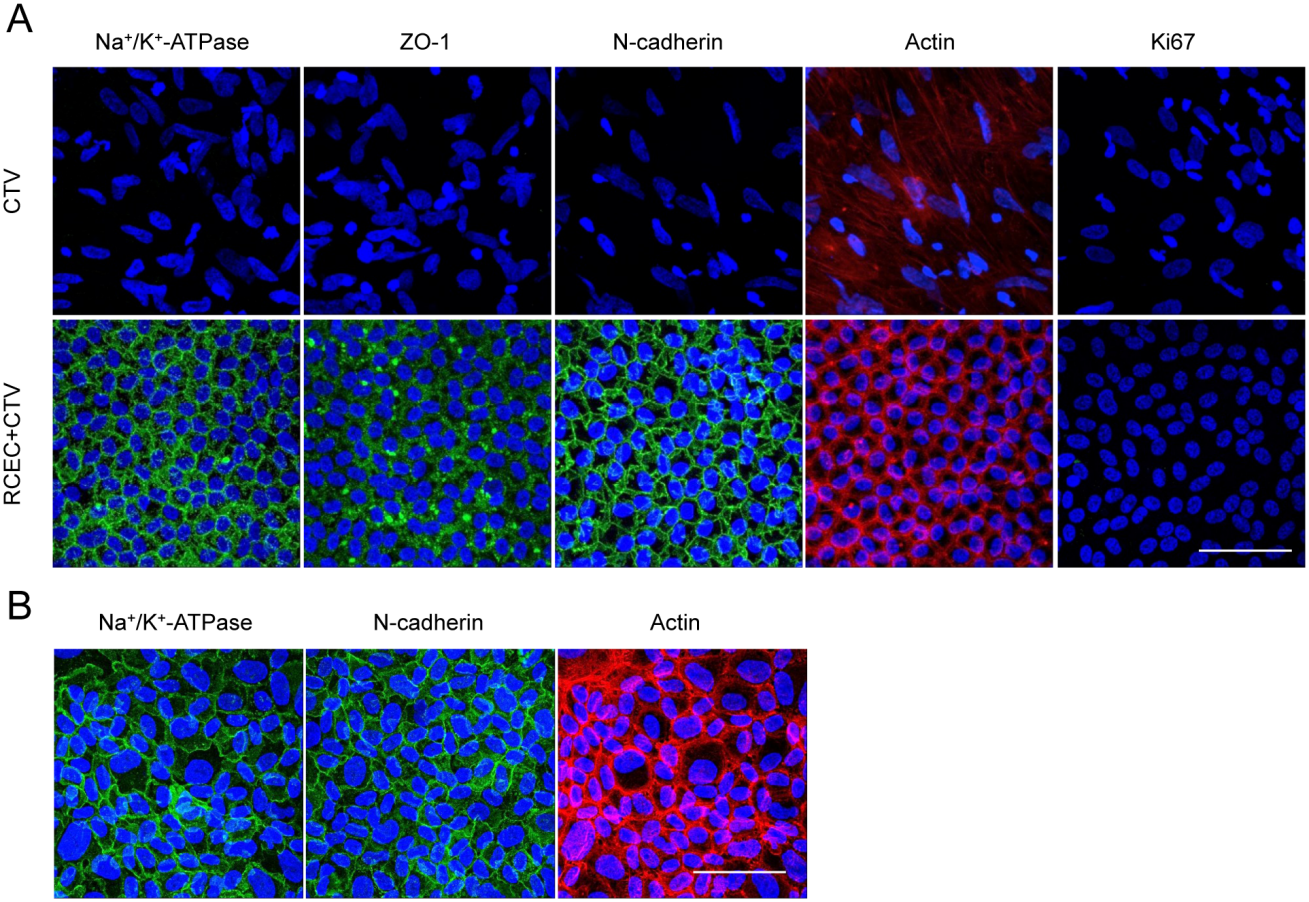
Histological assessment of corneal endothelium regenerated by injection of RCECs and HCECs after preservation in CTV. (A) Regenerated corneal endothelium was evaluated by immunofluorescent staining 2 weeks after cell transplantation. The function-related markers Na^+^/K^+^-ATPase, ZO-1, and N-cadherin were expressed in the corneal endothelium of the eyes injected with RCECs after 24 hours of preservation. Actin similar to that seen in healthy cells was observed along the cell cortex in the eyes injected with RCECs. Fibroblastic cells without function-related markers were observed. Scale bar: 50 μm. (B) Feasibility of using HCECs after preservation in CTV was evaluated by injecting HCECs preserved for 24 hours in CTV into the rabbit corneal endothelial dysfunction model (n = 3). Immunofluorescence staining showed that corneal endothelium was regenerated by the injected HCECs and expressed Na^+^/K^+^-ATPase and N-cadherin. Actin staining showed that the regenerated corneal endothelium in the rabbit eyes had a normal morphology consisting of a monolayer of hexagonal cells. Scale bar: 500 μm.

Immunofluorescent staining demonstrated that the function-related markers Na^+^/K^+^-ATPase, ZO-1, and N-cadherin were expressed in all regenerated CECs in eyes injected with preserved RCECs. The distribution of actin in the cell cortex was similar to that in healthy cells in the eyes injected with RCECs. On the other hand, control eyes, which showed fibroblastic morphology, showed no expression of function-related markers (Fig 4A).

We also investigated the feasibility of using HCECs after preservation in CTV. HCECs preserved for 24 hours in CTV were injected into the rabbit corneal endothelial dysfunction model. Immunofluorescent staining showed that the HCECs regenerated corneal endothelium that expressed function-related markers and showed a normal morphology (Fig 4B).

## Discussion

We recently obtained approval from the Japanese Ministry of Health, Labour, and Welfare to treat corneal endothelial dysfunction with cell-based therapy and started the first-in-human clinical trial in 2014 at Kyoto Prefectural University of Medicine (Clinical trial registration: UMIN000012534). This clinical trial uses cultured HCECs suspended in a vehicle consisting of modified OptiMEM-I supplemented with ROCK inhibitor, which are injected into the anterior chamber of the patients following removal of the diseased corneal endothelium. We are currently focusing our efforts on optimizing the original protocol of the principal investigator-initiated clinical trial to begin a sponsor- initiated clinical trial [20].

The use of intraocular irrigation solutions is allowed by regulatory authorities in intra ocular surgeries such as cataract surgery and vitreo-retinal surgery and are widely utilized. These intraocular irrigation solutions, which are combinations of buffers and antioxidants, were developed to mimic the composition of the aqueous and vitreous humor to maintain the integrity of the intraocular tissues. The physiological assessment of these solutions was focused on providing safety to the corneal endothelium, as damage to the corneal endothelium causes irreversible corneal haziness [21–23]. Intraocular irrigation solutions are assumed to be safe for cell-based therapy, but our current findings indicate that they hamper CEC adhesion onto substrates.

This finding suggests that an intraocular irrigation solution itself is unsuitable for use as a vehicle for cell-based therapy and that an optimized vehicle is important for the regeneration of the corneal endothelium. Our approach to generate a new vehicle was as follows: 1) screen the effects of various culture media on CEC adhesion, 2) generate a new vehicle by removing materials that are not favorable for clinical use, and 3) evaluate the feasibility of the vehicle in an animal disease model.

HCECs have limited proliferative ability and undergo transformation into a fibroblastic phenotype. Researchers have long recognized the difficulty of HCEC culture and have devoted their efforts to culture HCECs that can be used for research purposes and in regenerative medicine [17, 24–30]. Several culture media, such as F12, M199, SHEM, DMEM, and OptiMEM-I, were proposed as HCEC culture media when supplemented with materials such as serum and various growth factors [13, 24, 25, 27]. However, Zhu and colleagues, in a screening study of several culture media, demonstrated that OptiMEM-I promoted attachment and a moderate proliferative response [26]. We demonstrated the successful expansion of HCECs and suppression of fibroblastic transformation in an OptiMEM-I based culture medium [17, 19, 31]. A further screening of media modified from Eagle's minimum essential media (EMEM), like OptiMEM-I, showed that RELAR medium promoted the best HCEC adhesion among the media tested. However, media modified from EMEM, such as OptiMEM-I and RELAR, include hormones and growth factors, whereas EMEM contains amino acids, salts, glucose, and vitamins [32]. We generated CTV by removing hormones, growth factors, and potentially toxic materials to minimize the possible risk for the patients, although the amount of hormones and growth factors is probably sufficiently low to avoid causing adverse events such as carcinogenicity and other systemic disorders.

In the clinical setting, CECs will be cultured and harvested in a Good Manufacturing Practice (GMP) grade cell processing center (CPC), and will be transported to each facility. Thus, cell stability must be maintained for a certain time to enable transportation from the CPC to hospital. In this study, HCECs retained more than 85% viability after 24 hours of preservation in CTV, and the preserved RCECs and HCECs restored the corneal endothelium in the rabbit model of corneal dysfunction, confirming the efficacy of CEC preservation in CTV. However, possible adverse effects caused by dead cells generated during preservation should be carefully assessed.

Land et al. showed that human recombinant superoxide dismutase reduced the rejection episodes after renal allografts in a randomized double-blind trial [33]. They hypothesized that antioxidants suppressed the ischemia/reperfusion injury of the graft and then reduced the immunogenicity of the graft [33]. This phenomenon is currently explained by the concept of damage-associated molecular patterns (DAMPs) that trigger inflammatory responses against danger stimuli [34, 35]. The release of DAMPs by tissue stress or injury was confirmed to play an important role in the pathophysiology of a wide range of diseases [36–40]. In the current study, no adverse events, such as severe inflammation or rejection, were observed in rabbit model, but the possibility of an inflammatory response caused by CECs damaged during preservation should be further investigated.

In conclusion, we generated a CTV composed of amino acids, salts, glucose, and vitamins but without hormones, growth factors, and or potentially toxic materials for use for the treatment of corneal endothelial dysfunction using cell-based therapy. We also showed that CTV enabled the preservation of CECs for subsequent injection and that the preserved CECs restored a transparent cornea in rabbit model of corneal dysfunction. The current strategy for generation of a vehicle from a culture medium by removing materials that are unfavorable for clinical use is fast and practical and can be applied to cell-based therapy in other fields.

## Supporting Information

S1 File Participant-level data. This supporting information file provides participant-level data.
(XLSX)

S1 Table Composition table of cell therapy vehicle (CTV). This supporting table provides composition of CTV.
(PDF)
