# Supplementary material for: Generation and Feasibility Assessment of a New Vehicle for Cell-Based Therapy for Treating Corneal Endothelial Dysfunction
Source: PLoS One. 2016 Jun 29;11(6):e0158427. doi: 10.1371/journal.pone.0158427 (PMC4927169; doi:10.1371/journal.pone.0158427)
Supplement: S1 Table — This supporting table provides composition of CTV. (PDF) [file pone.0158427.s002.pdf]

S1 Table. Composition table of cell therapy vehicle (CTV)

Inorganic salts	Vitamins
$(NH_4)_6MO_7O_{24}\cdot 4H_2O$	Ascorbic acid 2-phosphate 3Na
$CaCl_2$	D-Biotin
$CuSO_4\cdot 5H_2O$	Choline Chloride
$FeSO_4\cdot 7H_2O$	Folic acid
$MgSO_4$	Folinic acid・Ca
$MnSO_4\cdot 5H_2O$	myo-Inositol
$NiCl_2\cdot 6H_2O$	Niacinamide
KCl	D-Pantothenic acid・1/2 Ca
$NaHCO_3$	Pyridoxal・HCl
NaCl	Riboflavin (B2)
$NaH_2PO_4\cdot H_2O$	Thiamine・HCl (B1)
Na_2SeO_3	Vitamin B12
Amino acids	DL-αTocopherol
L-Arginine ・HCl	Lipids
L-Aspartic acid	Arachidonic acid
L-Cysteine・HCl・H_2O	Cholesterol
L-Glutamic acid	Linoleic acid
L-Glutamine	Linolenic acid
Glycine	Myristic acid
L-Histidine・HCl・H_2O	Oleic acid
L-Isoleucine	Palmitic acid
L-Leucine	Stearic acid
L-Lysine・HCl	Deoxyribonucleosides
L-Methionine	Thymidine
L-Phenylalanine	Other components
L-Proline	D-Glucose
L-Serine	Disodium succinate
L-Threonine	HEPES
L-Tryptophan	Hypoxanthine
L-Tyrosine	Kolliphor P 188
L-Valine	Polyvinylpyrrolidone K90
	Putrescine・2HCl
	Sodium Pyruvate
	Succinic acid
	Tween 80
